# P-924. Infective Endocarditis Outcomes in Older Patients with HIV

**DOI:** 10.1093/ofid/ofae631.1115

**Published:** 2025-01-29

**Authors:** Aikaterini Papamanoli, Andreas P Kalogeropoulos, George Psevdos

**Affiliations:** Stony Brook University Hospital, Stony Brook, New York; Stony Brook University, Stony Brook, New York; Northport VA Medical Center, Northport, New York

## Abstract

**Background:**

Previous reports in patients with HIV (PWH) and infective endocarditis (IE) have reported variable mortality compared to their non-HIV counterparts. However, most studies are older, have included younger patients with high rates of IV drug use, and uncontrolled HIV status with low CD4 count. Contemporary antiretroviral therapy has stabilized HIV, but little is known about IE outcomes in the current PWH population, as underlying HIV-related inflammatory process may impact outcomes or HIV status may affect therapeutic approaches due to access issues or provider bias.

**Table 1.**
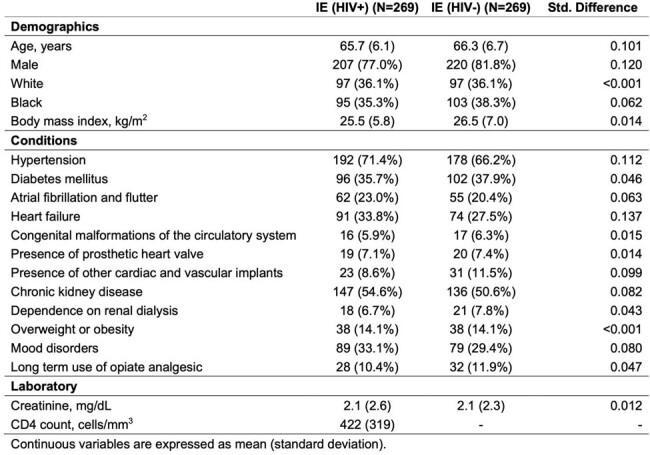
Baseline characteritics of the propensity matched cohorts.

**Methods:**

We used data from the TriNetX Research Network, an electronic health records network with over 100 million patients. We identified (1) a cohort of patients age ≥65 with HIV (ICD-10-CM code B.20) and IE (code I33.X) diagnosed between 1/1/2014 and 12/31/2023, and (2) a propensity score-matched cohort of patients age ≥65 with IE without HIV, matched for demographics, social history including substance abuse, and comorbid conditions. We evaluated 1-year rates of all-cause mortality, stroke, heart failure, and cerebral infections. We also evaluated the proportions of patients who underwent cardiac surgery in both groups. We used Cox proportional hazards regression to compare 1-year outcomes and logistic regression to estimate odds ratio for surgical treatment.

**Figure 1.**
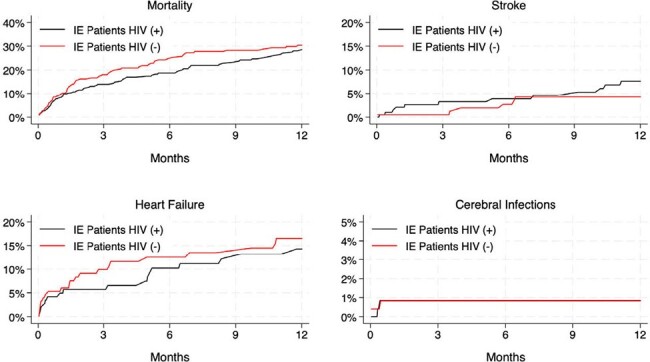
One-Year Kaplan-Meier rates of death, incident stroke, incident heart failure, and cerebral infections among patients with infective endocarditis and HIV (+) (black line) vs. HIV (-) (red line) status

**Results:**

We identified 269 PWH and IE and 269 matched patients with IE without HIV. The baseline characteristics of the cohorts were well balanced, **Table 1**. After 1-year, 68 (28.5%) patients in the PWH group had died vs. 69 (30.4%) in the non-HIV group (HR: 0.88, 95%CI 0.63 – 1.23; P=0.46). Both patient groups experienced similar 1-year rates of stroke (7.6% vs. 4.3%; P=0.23), heart failure (14.3% vs. 16.5%; P=0.55), and cerebral infections (0.8% vs. 0.8%; P=0.99), **Fig. 1**. Only 10 patients in each group (3.7% for both) underwent cardiac surgery (P >0.99).

**Conclusion:**

In contemporary older (age ≥65) PWH and IE, compared to matched patients without HIV and IE, no difference in 1-year mortality was observed. Other IE-associated outcomes were also similar. Interestingly, rates of cardiac surgery were similar but low in both groups. Therefore, our data suggest that possible HIV-related inflammation does not affect outcomes in IE among older patients with stable HIV status.

**Disclosures:**

**All Authors**: No reported disclosures

